# Bibliometrics Analysis of Research Progress of Electrochemical Detection of Tetracycline Antibiotics

**DOI:** 10.1155/2023/6443610

**Published:** 2023-02-18

**Authors:** Dihua Wu, Hassan Karimi-Maleh, Xiaozhu Liu, Li Fu

**Affiliations:** ^1^College of Materials and Environmental Engineering, Hangzhou Dianzi University, Hangzhou 310018, China; ^2^School of Resources and Environment, University of Electronic Science and Technology of China, P.O. Box 611731, Xiyuan Ave, Chengdu 610056, China; ^3^Department of Chemical Engineering and Energy, Laboratory of Nanotechnology, Quchan University of Technology, Quchan 94771-67335, Iran; ^4^Department of Chemical Sciences, University of Johannesburg, Doornfontein Campus, P.O. Box 17011, Johannesburg 2028, South Africa; ^5^Department of Cardiology, The Second Affiliated Hospital of Chongqing Medical University, Chongqing 400010, China

## Abstract

Tetracycline is a broad-spectrum class of antibiotics. The use of excessive doses of tetracycline antibiotics can result in their residues in food, posing varying degrees of risk to human health. Therefore, the establishment of a rapid and sensitive field detection method for tetracycline residues is of great practical importance to improve the safety of food-derived animal foods. Electrochemical analysis techniques are widely used in the field of pollutant detection because of the simple detection principle, easy operation of the instrument, and low cost of analysis. In this review, we summarize the electrochemical detection of tetracycline antibiotics by bibliometrics. Unlike the previously published reviews, this article reviews and analyzes the development of this topic. The contributions of different countries and different institutions were analyzed. Keyword analysis was used to explain the development of different research directions. The results of the analysis revealed that developments and innovations in materials science can enhance the performance of electrochemical detection of tetracycline antibiotics. Among them, gold nanoparticles and carbon nanotubes are the most used nanomaterials. Aptamer sensing strategies are the most favored methodologies in electrochemical detection of tetracycline antibiotics.

## 1. Introduction

Tetracycline antibiotics are a broad-spectrum class of antibacterial substances isolated from the actinomycete *Streptomyces aureofaciens*. The tetracyclines have been used for a long time, but they are still widely used today [1]. Tetracycline is used in the livestock and poultry industry to treat diseases such as intestinal infections. Making meat from livestock and poultry before the end of the rest period can result in tetracycline residues. Residues of tetracycline are now being found in meat and dairy products, including milk, honey and eggs [2]. When we consume these products daily, the residual antibiotics enter our body through the food chain and may cause allergic reactions or make our body resistant to the drugs. Tetracycline antibiotics are widely used in various fields because of their broad antibacterial spectrum and low cost. Because of the antibacterial properties of the antibiotics themselves, the ecological environment is seriously affected by tetracycline antibiotics [3]. When tetracycline is used in humans or animals, the drug is excreted as a prodrug or metabolite with the metabolism, and most of it enters the soil and water bodies. Under the action of various environmental factors, it can produce transfer, transformation or enrichment in plants and animals [4]. Whether in its original form or metabolites, the drug remains active during the migration process and can cause severe effects on microorganisms, aquatic organisms and insects. Only a small percentage of tetracycline is left in the animal's body after use. The toxicity of consuming this type of food does not manifest in a short period [5]. However, prolonged ingestion of food containing residual antibiotics can lead to various organ lesions due to accumulation effects. For example, tetracycline antibiotics can bind to calcium in the bones and have an inhibitory effect on the growth of human bones and teeth. Tetracycline taken orally by pregnant women in late pregnancy can also be deposited in the fetal dental tissues and affect the development of fetal milk teeth and permanent teeth. Therefore, long-term consumption of tetracycline can seriously affect human health [6].

So far, the detection methods of tetracycline include microbiological method, immunoassay, thin-layer chromatography, high-performance liquid chromatography (HPLC), liquid chromatography-mass spectrometry, spectroscopic analysis, and electrochemical method [7]. Different detection methods have different advantages and disadvantages, and their sensitivity and detection limits are also different. Among them, the microbiological method is currently recognized and widely used to determine the classical method of tetracycline antibiotic residues. This method is used to detect tetracycline antibiotics under mature conditions and with high accuracy. However, this assay is difficult to achieve the strong specificity and high accuracy required for the assay due to the lack of anti-interference ability [8]. At the same time, the method is complicated and time-consuming for the pre-treatment of the sample. HPLC has the advantages of high efficiency, rapidity, sensitivity and high detection rate, especially for the simultaneous detection of multiple substances. Therefore, HPLC is widely used in food hygiene departments to detect antibiotic residues in animal food [9].

In recent years, electrochemical analysis techniques have been used for the purpose of efficient long-term online monitoring of environmental pollutants [10–15]. Moreover, the simple principle of electrochemical detection, easy operation of the instrument and low cost of analysis and testing is widely used in pollutant detection. This method is characterized by high sensitivity, high accuracy and good selectivity, and the limit of detection of the measured substance can reach 10^−12^ M. Electrochemical analysis techniques have a series of different methodologies, including polarimetric analysis, molecular imprinting techniques and chemically modified sensors. For now, researchers are still searching for new, faster and more sensitive methods to detect tetracycline residues, such as immunosensors, enzyme sensors and aptamer sensors. So far, the electrochemical detection of tetracycline has been reviewed by several papers [16–18]. These reviews introduce the different methodologies and interpret the highlighted work. In this review, we attempt to analyze and review this topic statistically using a bibliometric approach. Bibliometric analysis is a literature and information mining method based on mathematical statistics. It can reflect research trends and hotspots through clustering relationships of keywords in the literature and has become an important tool for global analysis in various scientific fields [19–24]. This article hopes to analyze the collaborative networks and directions of investigation on this topic.

## 2. Data and Analysis Method

Two bibliometrics software have been used in this systematic literature review. The first is CiteSpace, developed by Dr. Chaomei Chen, a professor at the Drexel University School of Information Science and Technology [25–28]. CiteSpace 6.1R2 was used to calculate and analyze all documents. COOC is another emerging bibliometrics software [29]. COOC12.6 was used to analysis of country contribution and keywords co-occurrence. We used the core collection on Web of Science as a database to assure the integrity and academic quality of the studied material. “Tetracycline electrochemical sensor” or “tetracycline electrochemical detection” or “tetracycline electrochemical determination” has been used as a “Topic.” The retrieval period was indefinite, and the date of retrieval was June, 2022. 232 articles (including 5 early access) were retrieved (review and proceeding paper were not included in this survey).

## 3. Developments in the Research Field

### 3.1. Literature Development Trends

**Figure 1:**
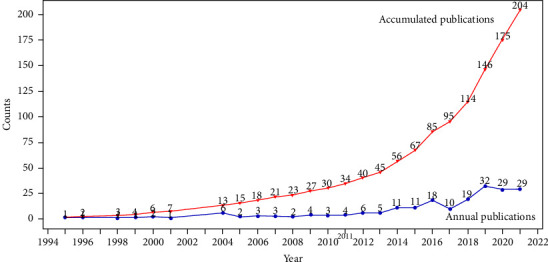
Annual and accumulated publications from 1995 to 2021 searched in the web of science about electrochemical detection of tetracycline antibiotics.

The number of papers published is an important indicator used to measure whether a topic is widely attracting the attention of scholars.  Figure 1 shows the annual and cumulative publications on electrochemical detection of tetracycline from 1995 to 2021. As can be seen, there were only sporadic reports on this topic before 2004. The earliest paper was published in 1995. Novaknepekli et al. [30] reported the preparation of doxycycline antibiotic sensors using a potential sensing strategy. In 1996, Tanase et al. [31] investigated the electrochemical reduction of tetracycline at a mercury drop electrode using alternating current polarography. They found that the reduction waves of tetracycline are very complex and that the electrolyte's concentration and pH significantly affect the assay results. In 1998, Tanase et al. [32] not only investigated minocycline by alternating current polarography but further employed voltammetry. Zhou et al. [33] investigated tetracycline, chlortetracycline and oxytetracycline antibiotics using capillary zone electrophoresis-rapid cyclic voltammetry in 1999. This early series of work investigated the electrochemical properties of tetracycline antibiotics. These results laid the foundation for later highly sensitive sensing assays for tetracycline antibiotics. From 2004–2013, this topic entered a period of steady development. This topic has been published every year, with the number of papers ranging from 2–6. This topic had its first growth between 2014–2017. The annual number of papers published in this period was more than 10. The second rapid growth in this topic started in 2018 and peaked at 32 papers in 2019. The annual number of papers published in both 2020 and 2021 is 29. As of July 2022, there have been 27 publications on this topic this year, representing another stable publication phase for this topic without a significant downward trend. This topic is now at the most active stage in its entire development history.

### 3.2. Journals, Cited Journals and Research Subjects

**Figure 2:**
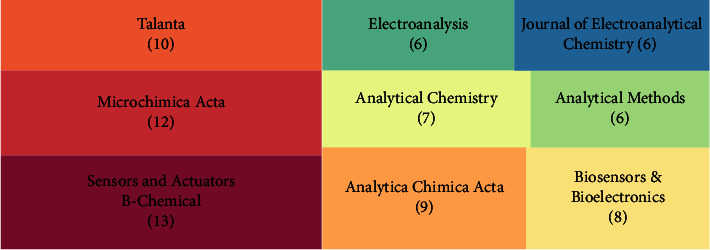
Top 9 journals that published articles on the electrochemical detection of tetracycline antibiotics.

Figure 2 shows a tree diagram of the top 9 journals publishing the number of electrochemical detection of tetracycline antibiotics. As seen from the figure, the journals that published the most papers on this topic all belonged to analytical chemistry, with Sensor and Actuators B-Chemical publishing the highest number of papers. Since this topic focuses on detecting tetracycline antibiotics using electrochemical techniques, the figure includes, in addition to traditional analytical chemistry journals, those focusing on electroanalytical chemistry, including Electroanalysis, Journal of Electroanalytical Chemistry, and Biosensors & Bioelectronics. Notably, all journals in this figure are classical journals in analytical chemistry and do not include journals launched in recent years. This represents that traditional analytical chemists favour the survey on this topic.

In addition to the number of papers published by the journal on the topic, the frequency with which the journal cited papers related to the theme is also an important indicator.  Table 1 shows the top 15 cited journals on the electrochemical detection of tetracycline antibiotics. Most of the journals in Figure 2 are included in Table 1, representing that they not only publish the most papers on this topic but are also most frequently cited in papers on this topic. Journals related to analytical chemistry, especially electroanalytical chemistry, continue to dominate Table 1. However, Table 1 also provides some additional information. This topic will also cover the Journal of Chromatography *A*, ranked eighth in Table 1, representing the chromatographic technique to detect tetracycline antibiotics. Based on our understanding of the field of electrochemical sensors, chromatography-related analytical techniques for the separation and detection of tetracycline are often used as a comparison to corroborate the performance of the proposed electrochemical sensors. In addition, Food Chemistry and Food Control appear in Table 1 to represent that the main application area for tetracycline assays is food quality control.

**Figure 3:**
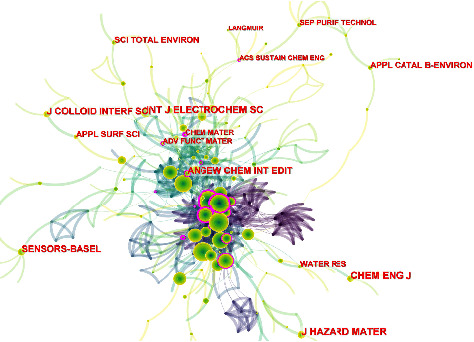
Co-occurrence network of cited journals for electrochemical detection of tetracycline antibiotics.

To further explore the information that journals provide, we constructed a co-occurrence network of cited journals related to the electrochemical detection of tetracycline antibiotics (Figure 3). In this figure, we have not labelled most of the journals discussed in Figure 2 and Table 1. The location at the centre of the figure contains the journals mentioned above. However, this co-occurrence figure provides some additional information.

1. In addition to some classic analytical chemistry journals, the International Journal of Electrochemical Science and Sensor-Basel also have a high frequency of appearances on this topic.
2. In the upper centre of the co-occurrence figure are two journals with high centrality (purple circles). There are Chemistry of Materials and Advanced Functional Materials, representing materials science is an important influence on this topic's development.
3. The periphery of the co-occurrence figure contains several journals focused on interface research, including Journal of Colloid and Interface Science, Applied Surface Science, and Langmuir. Signal changes in electrochemical sensors depend on the interface's chemical reactions. Therefore, it is important to investigate the nature of the interface for a sensor assembly.
4. The periphery of the co-occurrence figure also includes a series of journals related to environmental science, such as Applied Catalysis B: Environmental, Journal of Hazardous Materials, Water Research, etc. This represents the application area of this topic, in addition to the food presented in Table 1 and the environmental field.

Table 2 shows which journal was published for the first time on this topic in 2021 and 2022. As can be seen, a series of materials science-related journals appear. A series of materials science-related journals are starting to publish papers on this topic. This confirms the above speculation that materials science innovations significantly impact the performance of electrochemical sensors. In addition, some bio-related journals also appear in Table 2, representing the possibility that electrochemical sensing technology has been applied to detect tetracycline antibiotics in biological samples.

The category of the published paper can reflect the evolution of the topic.  Table 3 shows the evolution of the category of the electrochemical detection of tetracycline antibiotics over time. It can be seen that this topic in the early days involved mainly the fields of chemistry and biology. From 2009 onwards, categories related to materials science started to play an important role gradually. From 2012 onwards, categories of application areas related to tetracycline antibiotics are also included in the topic.

### 3.3. Geographic Distribution

**Figure 4:**
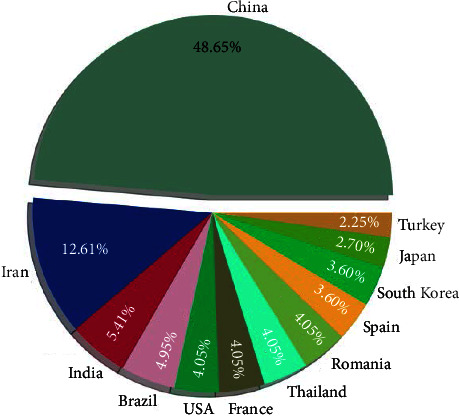
Pie chart of papers related to electrochemical detection of tetracycline antibiotics contributed by different countries.

Figure 4 shows the top 12 countries with the most publications on electrochemical detection of tetracycline antibiotics. China contributed the most significant number of papers, at 48.65%. The fact that China has published nearly half of the papers on this topic can be attributed to three reasons. First, China has a large community of scientific and technical personnel and therefore plays an important role in academic research. Second, electrochemical analysis is a field with a long history in China. It has a very broad market in China for commercial products. Finally, tetracycline is widely used in China's farming industry, making the environmental pollution it causes a significant challenge. Iran and India also play an essential role in this topic, contributing 12.61% and 5.41% of the papers, respectively. Brazil, USA, France, Thailand and Romania contributed more than 4% of the papers. As seen from the figure, this theme has attracted much attention in Asia, probably because Asian countries have been using many tetracycline antibiotics. At the same time, the topic has attracted several countries in South America and Europe due to the widespread use of tetracycline antibiotics worldwide.

**Figure 5:**
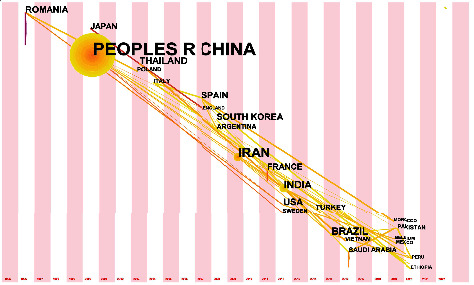
Time-zone view of geographic distribution for electrochemical detection of tetracycline antibiotics (The year of the node in the graph is the time when a paper on this topic was first retrieved by a particular category in WOS. The size of a node is proportional to the number of connections between this paper and other nodes).

Figure 5 shows the time-zone view of the geographic distribution for electrochemical detection of tetracycline antibiotics. Links between countries are established based on papers published directly cited in those countries. China was not involved at the beginning of this topic. Hungary, Romania and Canada conducted pre-investigations on this topic before 2000. Starting in 2000, China and Japan joined the survey on this topic. Between 2007 and 2012, a range of countries participated in this topic, including Spain, South Korea, Argentina, Iran, India and France. Starting in 2016, a series of additional countries began to participate in this topic, including Brazil, Saudi Arabia, Pakistan and Vietnam. This trend is directly correlated with the two increases in the number of papers published in Figure 1.

**Figure 6:**
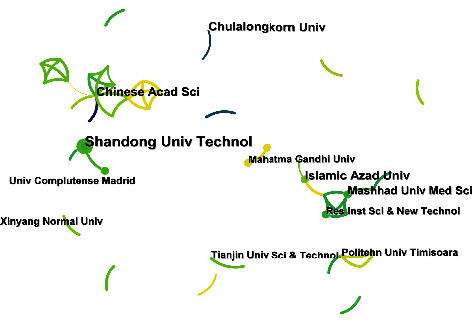
Institution cooperation network for electrochemical detection of tetracycline antibiotics.

Although many countries are involved in this topic, it does not form a very complex network of cooperation.  Figure 6 shows the institutional cooperation network for this topic. It can be seen that this topic has formed 2 main collaborative networks so far. The first collaborative network is led primarily by the Chinese Academy of Sciences and includes a range of Chinese universities and research institutions. In addition, Pakistani universities are involved in this collaborative network, including The Government College University, Lahore and COMSATS University Islamabad. The second collaborative network is led by Mashhad University of Medical Sciences, Islamic Azad University, and Research Institute of Sciences and New Technology. This collaborative network is composed of Iranian research institutions and universities. This shows that the pattern of collaboration on this topic is domestic and does not result in extensive international collaboration. This may be because tetracycline contamination and the strategies to cope with it are different in each country, making it difficult to conduct investigations based on the same purpose.

## 4. Keyword Analysis and Evolution of the Field

The most effective way to understand the direction of investigating concerns in a topic is the analysis of keywords.  Table 3 lists the top 15 keywords in this topic. Since this topic is about the electrochemical detection of tetracycline, the most frequent keyword is related to antibiotics and electrochemistry. In addition, some other keywords provide information on the different research directions on this topic. For example, milk is ranked 5th in Table 4 with a total of 32 occurrences, representing that it is the most frequently used real sample for detecting tetracycline. The quality of milk is extremely important, but the reality is that cows are susceptible to mammary gland disease, which can significantly decline some milk quality [34]. To avoid this quality risk, some dairy farmers inject their cows or add antibiotics to their feed to prevent them from contracting diseases [35]. Tetracycline is widely overused because of its good antibacterial effect and low price. Cows are usually treated with intramuscular or intravenous injections [36]. After circulation, the antibiotics end up in the udder, which can quickly end up in the milk. In other cases, injections are also given directly into the cow's lesion, a method that is more likely to produce antibiotic residues in the milk. If cow's milk or dairy products containing antibiotic residues reach the market, people unknowingly drink them to accumulate low doses of antibiotics [37]. The beneficial bacteria in the human intestine will be affected by the ingested antibiotics, giving room for the growth of pathogenic bacteria and causing local or even systemic infections in the body. In addition, water also appears in Table 4, representing the importance of tetracycline detection in water bodies. The primary sources of tetracycline antibiotics in the environment include industrial effluents, farming antibiotics, and medical antibiotics [38].

Nanoparticle and gold nanoparticle are ranked 6th and 12th respectively in Table 4, representing that nanomaterials significantly influence this topic. In the last section of the journal analysis, we found a series of material science journals appearing on this topic, representing the synthesis and application of new materials that can improve the performance of electrochemical sensors [39]. Among them, metallic nanomaterials, especially noble metal nanomaterials, are most widely used in analytical assays. For example, nano gold and nano platinum have good biocompatibility and can maintain the activity of enzymes [40]. Carbon nanotubes also appear in Table 4, representing that it is also widely used as a material for electrochemical sensor preparation in this topic. Carbon nanotubes are seamless tube-like, quasi-one-dimensional carbon materials with nanoscale diameters formed from convoluted graphene sheets. The carbon atoms in the tubes are mainly bonded by sp^2^ hybridization to form hexagonal lattice-like graphene sheets. Carbon nanotubes can be classified into single-walled carbon nanotubes (SWCNTs) and multi-walled carbon nanotubes (MWCNTs), depending on the number of carbon atom layers. Carbon nanotubes are widely used in electrochemistry because of their large specific surface area and low resistivity and are considered excellent materials for nanodevices and interconnect devices [41].

Another important keyword in Table 4 is aptasensor. The aptamer is a class of single-stranded oligonucleotides (DNA, RNA and modified RNA) that can be synthesized by exponentially enriched ligand phylogenetic techniques (SELEX). It possesses the affinity to bind specifically to the corresponding target molecule [42]. The specific binding of the aptamer to the corresponding target molecule is based on the diversity of single-stranded nucleic acid structures and their spatial conformations. Compared to common chemical antibodies, aptamers have an advantage in specificity and selectivity. In addition, the aptamer is synthesized in vitro and a shorter cycle, unlike antibody preparation which takes at least five or six months [43]. At the same time, the aptamer is chemically stable, such as a certain degree of thermal complexation, and easy to preserve. More importantly, various groups can be modified on the aptamer as needed, such as functionalized groups like sulfhydryl, amino, and hydroxyl groups. Their application with nanomaterials such as nanogold can be combined into Au-S bond, Au-NH_2_ bond, etc., which facilitates the effective immobilization of aptamers [44]. Therefore, aptasensor is a detection method that combines highly sensitive sensor technology with aptamer and target detector specific response, which has the advantages of both high selectivity of aptamer analysis and high sensitivity of the electrochemical analysis.

**Figure 7:**
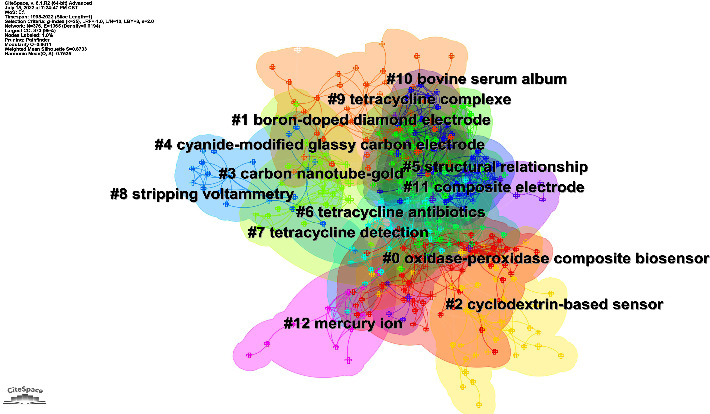
Grouping of keywords for electrochemical detection of tetracycline antibiotics.

Cluster analysis can further understand the different directions of investigation in this topic.  Figure 7 shows that 13 clusters were formed after clustering the keywords. On the whole, many clusters have overlapping areas between them, indicating that their contents have more similarities with each other.  Table 5 describes the clusters and their ID, size (number of papers), silhouette, and respective keywords.

## 5. Conclusion and Perspectives

Nowadays, the main detection techniques for tetracycline antibiotics are HPLC, HPLC/MS, UV and fluorescence methods. Although the detection limits of these methods can meet the experimental requirements, the equipment costs are expensive, the sample analysis methods are complicated, and the real-time monitoring of pollutants cannot be achieved. Therefore, it is necessary to develop simple, inexpensive, fast and efficient electrochemical sensors with high accuracy. This review provides a bibliometrically based review of the development of electrochemical detection of tetracycline antibiotics from 1995–2022. The statistical analysis led to the following conclusions:

1. Studies on electrochemical detection of tetracycline antibiotics have been reported since 1995 but did not receive much attention immediately. Papers on this topic started to receive gradual attention in 2004 and entered a period of rapid growth in 2014.
2. The investigation of this topic has attracted many scholars from Asia and South America. Among them, China, Iran and India have contributed a large number of papers on this topic. However, this topic has not resulted in extensive international collaboration.
3. Papers on this topic are mainly published in classical analytical chemistry, especially in journals related to electrochemistry. Materials science-related journals have also started to publish papers on this topic in recent years. Developments and innovations in materials science can enhance the performance of electrochemical detection. Among them, gold nanoparticles and carbon nanotubes are the most used nanomaterials to enhance electrochemical sensing performance.
4. Although tetracyclines are electrochemically active and can be oxidized and reduced on common electrode surfaces. However, more sensitive and selective detection of tetracycline antibiotics can be achieved using aptamers.
5. The main application scenarios for electrochemical detection of tetracycline are the detection of water and food (especially milk).

Meanwhile, based on the review of this topic, we believe that the following issues need to be investigated regarding the electrochemical detection of tetracycline:

1. Samples containing tetracycline often contain other substances, so the anti-interference of electrochemical detection techniques is a challenge that needs to be addressed.
2. How to improve the detection efficiency of electrochemical sensors is also an important challenge. Conventional reusable electrodes can become contaminated during testing. Therefore, how to regenerate the electrode or improve the service life is also very important.
3. Current electrochemical sensors still rely on sampling a sample. How to achieve instant detection is also a future challenge.

## Figures and Tables

**Table 1:** Top 15 cited journals on the electrochemical detection of tetracycline antibiotics.

No.	Citation	Cited journal
1	147	Sensors and actuators B: chemical
2	139	Biosensors and bioelectronics
3	137	Talanta
4	134	Analytica chimica acta
5	119	Analytical chemistry
6	93	Electrochimica acta
7	92	Journal of electroanalytical chemistry
8	90	Journal of chromatography a
9	83	Analyst
10	81	Food chemistry
11	81	Electroanalysis
12	79	Analytical and bioanalytical chemistry
13	68	Microchimica acta
14	66	Food control
15	62	Journal of the American chemical society

**Table 2:** List of journals has published paper for electrochemical detection of tetracycline antibiotics in the last two years.

Year	Journals
2022	Applied nanoscience; diamond and related materials; environmental pollution; inorganic and nano-metal chemistry; journal of cleaner production; journal of materials science; journal of materials science-materials in electronics; journal of molecular liquids; journal of photochemistry and photobiology a-chemistry; journal of solid state chemistry; Korean journal of chemical engineering; spectrochimica acta part a-molecular and biomolecular spectroscopy
2021	Acs applied materials & interfaces; adsorption science & technology; biotechnology and applied biochemistry; chemosensors; international journal of environmental analytical chemistry; journal of fluorescence; journal of food measurement and characterization; journal of physical chemistry c; journal of sensors; nanomaterials; optical materials; polymer bulletin

**Table 3:** Research categories for electrochemical detection of tetracycline antibiotics.

Year	WoS categories
1995	Chemistry, multidisciplinary
1996	Chemistry, analytical
1999	Biochemical research methods
2004	Pharmacology & pharmacy
2007	Electrochemistry; instruments & instrumentation
2008	Biochemistry & molecular biology; chemistry, medicinal; chemistry, organic;
2009	Nanoscience & nanotechnology; materials science, multidisciplinary; food science & technology; chemistry, physical; chemistry, applied; physics, atomic, molecular & chemical; agriculture, multidisciplinary
2010	Engineering, chemical; biotechnology & applied microbiology
2011	Physics, applied
2012	Environmental sciences; engineering, environmental; materials science, coatings & films
2013	Biophysics
2014	Polymer science; engineering, multidisciplinary; mineralogy
2015	Spectroscopy; engineering, electrical & electronic
2017	Multidisciplinary sciences; materials science, biomaterials; energy & fuels; thermodynamics
2018	Materials science, textiles; endocrinology & metabolism; toxicology
2019	Nutrition & dietetics; biology; microbiology
2020	Physics, condensed matter; acoustics
2021	Optics
2022	Chemistry, inorganic & nuclear; green & sustainable science & technology

**Table 4:** List of top 20 keywords for electrochemical detection of tetracycline antibiotics.

No.	Freq	Centrality	Keyword
1	61	0.36	Antibiotics
2	57	0.14	Residue
3	40	0.05	Tetracycline
4	39	0.09	Sensor
5	32	0.12	Milk
6	31	0.04	Nanoparticle
7	29	0.14	Electrode
8	26	0.03	Liquid chromatography
9	25	0.16	Oxytetracycline
10	23	0.21	Electrochemical detection
11	23	0.10	Biosensor
12	22	0.04	Gold nanoparticle
13	22	0.03	Electrochemical aptasensor
14	21	0.07	Electrochemical sensor
15	19	0.03	Aptasensor
16	18	0.18	Performance liquid chromatography
17	17	0.08	Degradation
18	13	0.05	Fabrication
19	12	0.05	Water
20	12	0.07	Carbon nanotube

**Table 5:** Knowledge clusters information of electrochemical detection of tetracycline antibiotics (*g*-index: *k* = 20; pruning method: pathfinder + pruning sliced networks + pruning).

Cluster ID	Size	Silhouette	Keywords	References
0	51	0.828	Nanoparticle; electrode; electrochemical sensor; fabrication; water; carbon nanotube; antibiotic residue;	[45–69]
1	44	0.740	Gold nanoparticle; electrochemical aptasensor; aptamer; composite; amplification	[49, 50, 70–84]
2	40	0.926	Acid; adsorption; extraction; reduced graphene oxide	[56, 61, 85–97]
3	31	0.925	Sensor; liquid chromatography; biosensor; performance	[50, 53, 72, 89, 98–106]
4	29	0.818	Oxytetracycline; probe; chlortetracycline; fluorescence detection	[76, 107–114]
5	25	0.967	Degradation; assay; amperometric detection; waste water	[59, 75, 115–122]
6	24	0.820	Milk; capillary electrophoresis; separation; glassy carbon electrode;	[33, 54, 70, 73, 107, 123–130]
7	24	0.851	Antibiotics; residue; tetracycline; DNA aptamer;	[32, 51, 52, 71, 87, 88, 99, 111, 124, 131–151]
8	21	0.939	Ascorbic acid; electrochemical immunosensor;	[133, 152, 153]
9	20	0.880	Performance liquid chromatography; solid phase extraction; nanocomposite; mass spectrometry;	[108, 110, 131, 154–159]
10	18	0.952	Electrochemical detection; modified electrode;	[64, 140, 160–163]
11	16	0.853	Electrochemical determination; film; sample; quantum dot;	[46, 51, 136, 138, 164–167]
12	12	0.976	Aptasensor; sensitive detection; ultrasensitive detection;	[168–175]

## Data Availability

No data were used to support this study.
